# Editorial: Social Interaction in Animals: Linking Experimental Approach and Social Network Analysis

**DOI:** 10.3389/fpsyg.2017.00035

**Published:** 2017-01-19

**Authors:** Cédric Sueur, Frédéric Mery

**Affiliations:** ^1^Centre National de la Recherche Scientifique (CNRS), Institut Pluridisciplinaire Hubert Curien UMR 7178, Université de StrasbourgStrasbourg, France; ^2^Evolution, Génomes, Comportement and Ecologie, Centre National de la Recherche Scientifique (CNRS), Institut de Recherche pour le Développement (IRD), Université Paris-Sud, Université Paris-SaclayGif-sur-Yvette, France

**Keywords:** fitness, information, social transmission, health, disease, social structure, RSiena

Understanding the link between individual behavior and population organization and functioning has long been central to ecology and evolutionary biology (Krause et al., 2009; Sueur et al., 2011; Kurvers et al., 2014). Behavior is a response to intrinsic and extrinsic factors including individual state, ecological factors, or social interactions. Within a group, each individual can be seen as part of a network of social interactions varying in strength, type, and dynamic. The structure of this network can deeply impact the ecology and evolution of individuals, populations, and species.

Three studies in this present issue tried to understand how group members are socially structured in non-human primates. Borgeaud et al. used a stochastic actor-oriented model (RSiena Package, Snijders, 2001) to test the dynamics of relationships of three groups of wild vervet monkeys. They found that triadic closure was significant in all three groups while degree popularity was significant in only two groups. Moreover, the dynamics of relationships according to the attributes of sex, matriline, and age differed significantly among groups.

In another way, Sosa showed that in Barbary macaques, females are more central, more active, and have a denser ego network in the social network than males; thus, they contribute in a greater way to the cohesive structure of the network. High-ranking individuals are likely to receive fewer agonistic behaviors than low-ranking individuals, and high-ranking females receive more allogrooming. Revealing the positions, the roles, and the interactional behavioral patterns of individuals can help understand the mechanisms that shape the overall structure of a social network.

Naud et al. studied another species of primates, the Mandrills. The objective of their study was to investigate how the group spatial distribution of a semi-free ranging colony of Mandrills in a food competition context relates to its social organization. Their results showed that high-ranking individuals were more observed in proximity of the feeding zone but that affiliative relationships were also associated with individual spatial distributions and explain more the individual distribution than dominance hierarchy.

These studies showed that within a group social interactions can take many forms and may significantly affect an individual's fitness (Silk et al., 2003; Formica et al., 2012; Kurvers et al., 2014). These interactions may result in complex systems at the group-level, such as in the case of collective decisions (Sueur et al., 2012). Among them, social transmission of information has been studied mostly in vertebrates (Whiten and van Schaik, 2007). Duboscq et al. reviewed the context and the methodology of experiments testing social transmission of information. However, they also discussed the reasons why social transmission sometimes does not occur despite being expected to and spanned a full range of mechanisms and processes including the constraints imposed by the social networks in which animals are embedded.

In a study on zebra finches, Fernandez et al. designed a method analyzing group vocal network semi-automatically. They wanted to test the hypothesis that the social structure of the group influences the parameters of the group vocal network. Using Markov analysis and cross-correlation analyses, they showed that juveniles as well as adults were more likely to respond to individuals of their own age-class.

In insects, social learning has been unambiguously demonstrated in social Hymenoptera but this probably reflects limited research effort and recent evidence show that even non-eusocial insects such as Drosophila, cockroaches, and crickets can copy the behavior of others (Battesti et al., 2012, 2015; Waters and Fewell, 2012). In this way, Pasquaretta et al. also used the RSiena package to analyze the dynamic of the interaction network of the fruit fly *Drosophila melanogaster* during social learning experiments. This work showed the importance of new methodologies in social network analyses to better understand causes and effects of animal social networks properties. The study of the processes which may facilitate or prevent this transmission and the analyses of the relationship between social network structure and efficiency of social transmission became in recent years an emerging and promising field of research (Sueur, 2011; Pasquaretta et al., 2014).

For instance, a number of recent studies have used Network Based Diffusion Analysis (NBDA) to detect the role of social transmission in the spread of a novel behavior through a population (Franz and Nunn, 2009; Hoppitt et al., 2010). Whalen and Hoppitt presented in this special issue a unified framework for performing NBDA in a Bayesian setting, and demonstrated how the Watanabe Akaike Information Criteria (WAIC) can be used for model selection. They performed a large scale simulation study and found that NBDA using WAIC could recover the correct model of social transmission under a wide range of cases, including under the presence of random effects, individual level variables, and alternative models of social transmission.

On another topic, Senior et al. worked on an integrated model approach between social network analysis and nutritional behavior. Animals have evolved complex foraging strategies to obtain a nutritionally balanced diet and associated fitness benefits. This nutritional behavior can also influence animal social interactions and affect group structures. Senior et al. demonstrated how social network analyses can be integrated into such a nutritional modeling framework. They illustrated their approach by examining the case of nutritionally mediated dominance hierarchies and demonstrated how metrics from social network analyses can be used to predict the fitness of agents in these simulations.

Health is a component of fitness also very well studied in Animal Behavioral Sciences (Abbot et al., 2011; MacIntosh et al., 2011; Rico-Uribe et al., 2016). In their study, McCowan et al. argued that nonhuman primate social systems are sufficiently complex to serve as model systems to study links between social life and health as we might observe in Humans. The influence of social contexts influencing health and fitness in non-human primates might help us to improve human health.

Finally, Golemiec et al. used a layer motif approach to understand social networks of kindergarten children and concluded that this method can be applicable on a more general scale to any group of individuals where interactions and identities can be readily observed and scored.

Using different animal species, including humans, this special issue investigated and showed how the structure of a group affects social interaction, information transfer, and collective decisions; but also how individuals treat different sources of information according to their sociality and the latest methodologies used to understand these processes.

## Author contributions

All authors listed, have made substantial, direct and intellectual contribution to the work, and approved it for publication.

## Funding

This project is funded by an ANR programme Blanc (ANR 12 BSV7 0013 02) to FM and CS and to an ANR Programme Santé Publique (ANR-15-CE36-0005-01) to CS. CS is funded by the University of Strasbourg Institute for Advanced Study (USIAS).

### Conflict of interest statement

The authors declare that the research was conducted in the absence of any commercial or financial relationships that could be construed as a potential conflict of interest. The reviewer DS and handling Editor declared their shared affiliation, and the handling Editor states that the process nevertheless met the standards of a fair and objective review.
